# The Effect of Laterality in Modified Radical Neck Dissection on the Risk of Intraoperative Bradycardia: A Retrospective Cohort Study

**DOI:** 10.7759/cureus.50129

**Published:** 2023-12-07

**Authors:** Giakoumis Mitos, Soteris Soteriades, Zisis Katsakis-Giannakis, Meltem Perente, Giannoula Thoma, Despoina Sarridou

**Affiliations:** 1 Anesthesiology, Critical Care and Pain, AHEPA University Hospital, Aristotle University of Thessaloniki, Thessaloniki, GRC; 2 Ansethesiology, George Papanikolaou General Hospital, Thessaloniki, GRC; 3 Anesthesiology, George Papanikolaou General Hospital, Thessaloniki, GRC; 4 Intensive Care Unit, St. Paul General Hospital, Thessaloniki, GRC; 5 Anaesthesiology, AHEPA University Hospital, Aristotle University of Thessaloniki, Thessaloniki, GRC

**Keywords:** cohort study, retrospective study, intraoperative complication, bradycardia, radical neck dissection

## Abstract

Background

Intraoperative bradycardia is a hardly studied complication of modified radical neck dissection (MRND).

Methods

Using convenient sampling, we retrospectively studied a cohort (n = 159) of patients who underwent MRND at Papanikolaou General Hospital, Thessaloniki, Greece between 2019 and 2020 to investigate whether MRND laterality (bilateral vs. unilateral) affects the occurrence of intraoperative bradycardia (a pulse rate lower than 50 bpm).

Results

Roughly two-thirds of the patients underwent unilateral MRND, and the rest underwent bilateral MRND. Bradycardia was observed in 25.8% of the cohort. We used logistic regression and investigated several potential confounding factors. Unilateral MRND was associated with a lower risk of intraoperative bradycardia compared to bilateral MRND in the simple regression model (relative risk (RR): 0.555, 95% confidence interval (CI): 0.331-0.932, p = 0.027). MRND laterality was not significantly associated with intraoperative bradycardia (p = 0.082) in the multiple regression model, whereas an American Society of Anesthesiologists physical status (ASA-PS) score of 3 vs. 4 (adjusted odds ratio (aOR) = 0.125, 95% CI: 0.0340-0.457, p = 0.002), the presence of atrial fibrillation (aOR = 11.4, 95% CI: 4.10-31.8, p < 0.001) and induction of anesthesia with dexmedetomidine (aOR = 4.57, 95% CI: 1.34-15.6, p = 0.015) were significantly associated with intraoperative bradycardia.

Conclusions

MRND laterality was close to statistical significance. Bigger sample sizes may provide more definitive information since the effect of MRND laterality on intraoperative bradycardia remains unclear. Our findings can inform clinical practice so that clinicians know when to expect bradycardia and are better prepared to manage it.

## Introduction

Radical neck dissection is a century-old operation that aims to remove cervical lymph nodes and other structures in the neck (muscles, vessels, nerves, salivary glands) in patients with various types of head and neck cancer in order to prevent metastatic spread [1]. In modified radical neck dissection (MRND), at least one non-lymphatic structure of the neck is spared while cervical lymph nodes are still resected. MRND may be unilateral or bilateral. There are several potential intraoperative complications associated with MRND. Possibly one of the most underestimated complications is bradycardia, which is linked to stimulation of the carotid sinus [1,2]. The carotid sinus is a bulge in the internal carotid artery just above the common carotid bifurcation, arising from baroreceptors in the artery adventitia. When carotid artery pressure increases, the carotid sinus stimulates parasympathetic outflow, leading to vasodilation, as well as a negative chronotropic and inotropic effect on the heart itself [3,4]. The carotid sinus may become hypersensitized due to pressure exerted by the tumor itself, by surgical manipulation, or by intra- and post-operative edema. Carotid sinus hypersensitization leads predominantly to cardioinhibition (decreased heart rate) but may also cause vasodilation or a mixed effect [5,6]. This retrospective cohort study is the first of its kind, as we aim to explore the effect of the laterality of MRND on the risk of intraoperative bradycardia. Furthermore, we aim to explore confounding factors contributing to bradycardia. For this reason, we involved patients undergoing MRND in Thessaloniki, Greece between 2019 and 2020. The identification of risk factors for intraoperative bradycardia may lead to modification of the surgical technique as well as changes in anesthetic management in order to prevent or treat bradycardia more efficiently.

## Materials and methods

The population being studied is patients undergoing elective MRND. A convenient sample of all patients that had undergone MRND at “G. Papanikolaou” General Hospital in Thessaloniki, Greece between January 1, 2019 and December 31, 2020, was selected. Patients undergoing other types of neck dissection, patients with sick sinus syndrome, patients with heart block, and patients with pacemakers were excluded from this study. Patients with missing values were also excluded. No patient received a local injection of lidocaine in the carotid bulb.

Power analysis

We were unable to find previous studies with similar measurements. One study [3] recorded the occurrence of a vasovagal reflex, defined as a drop-in pulse rate (PR) by more than 25 bpm or to a level below 60 or a drop in both systolic (SBP) and diastolic blood pressure (DBP) of at least 20 mmHg. The incidence was 36.8%. We hypothesized that, due to using a different definition of bradycardia (heart rate below 50 bpm), the total incidence in our study would be about 20%. It was decided that a clinically important difference would be if the group undergoing bilateral MRND had double the incidence of intraoperative bradycardia. Therefore, the incidence in each group (bilateral vs unilateral MRND) would be 26.7% vs 13.3%, respectively, assuming equal group sizes. Based on these assumptions and using a type I error probability α = 0.05 and a type II error probability β = 0.2, the power calculation method that we used [7] produced a minimum sample size required to identify such a difference is 137 patients.

Exposure variables

Information was collected on several exposure variables. The main exposure variable under investigation was the laterality of MRND, i.e., whether it was unilateral or bilateral. Several variables were explored as potential confounders. The following were used as binary (nominal) variables: sex, presence of the various diseases (hypertension, diabetes mellitus (DM), coronary artery disease (CAD), atrial fibrillation (AF), cerebrovascular disease (CVD), hypothyroidism, glaucoma), certain regular medication (ipratropium, beta-blockers, angiotensin-receptor blockers (ARB), angiotensin-converting enzyme inhibitors (ACEI), renin inhibitors, calcium channel blockers (CCB), antidepressants, amiodarone), whether the patient received dexmedetomidine for anesthesia induction and the drugs used for maintenance of anesthesia (sevoflurane, desflurane, propofol, and remifentanil). The American Society of Anesthesiologists Physical Status (ASA-PS) classification score was used as an ordinal variable. Age, body mass index (BMI), SBP and DBP, and the PR before anesthesia induction, the total dose of fentanyl received intraoperatively as well as the total duration of surgery and anesthesia were used as ratio-type continuous variables.

Outcome variable

We defined intraoperative bradycardia as a drop of the PR, as measured by the anesthesia monitor, to any level below 50 bpm for any length of time between the onset of anesthesia and the end of the operation.

Statistical analysis

Initially, we divided the data based on MRND laterality (unilateral vs. bilateral) to perform a frequency analysis for each categorical variable under investigation and provide descriptive statistics for quantitative variables. In the case of quantitative variables, we tested for normality using the Shapiro-Wilk test. For normally distributed data, we reported the mean and standard deviation (SD), whereas for non-normally distributed data, we reported the median, interquartile range (IQR), minimum and maximum value. We then developed simple univariate models for the association between each exposure variable and intraoperative bradycardia. For categorical exposure variables, we used Pearson’s chi-squared test and for quantitative variables, we used simple logistic regression. Both the odds ratio (OR) and the relative risk (RR) were reported for models with categorical exposure variables. If the p-value for any association was < 0.2, then this was included in the multiple univariate models, in which multiple logistic regression was used to account for potential confounders. Intraoperative bradycardia remained the outcome variable. The exposure variables included in this model were manually eliminated in a backward stepwise fashion (based on p-value), in order to arrive at the final adjusted model. The final model only contained the main exposure variable (regardless of the statistical significance of its correlation with the outcome) and all confounding exposure variables with p-values less than 0.05. Once the exposure variables of the final model were determined, potential interactions were investigated using an EVW hierarchical model, but none of the interactions were statistically significant. Therefore, they were not included in the final adjusted model.

The two-tailed level of statistical significance was set at 0.05 for all tests used above. 95% confidence intervals (CI) are provided for each point estimate. IBM® SPSS® Statistics for Windows, Version 29.0 (IBM Corp., Armonk, NY, USA) was used for the statistical analysis.

Ethical approval

Data were anonymized and ethical approval for the study was obtained by the study authors from the Scientific Council of the “G. Papanikolaou” General Hospital of Thessaloniki on January 15, 2020 (approval ID: 36/14.1.2020).

## Results

Descriptive statistics

A total of 159 patients were included in this study. Initially, we present a frequency analysis in Table 1 for all categorical data.

**Table 1:** Frequency analysis for all categorical data in patients undergoing unilateral or bilateral modified radical neck dissection (MRND). Values are absolute numbers and proportions expressed as percentages in brackets.

Variable	Value	Unilateral MRND n = 104	Bilateral MRND n = 55
Sex	Female	52 (50.0%)	21 (38.2%)
ASA-PS	2	4 (3.8%)	1 (1.8%)
	3	93 (89.4%)	46 (87.4%)
	4	7 (6.7%)	8 (14.5%)
Hypertension	Yes	66 (63.5%)	36 (65.5%)
Diabetes mellitus	Yes	18 (17.3%)	5 (9.1%)
Coronary artery disease	Yes	7 (6.7%)	5 (9.1%)
Atrial fibrillation	Yes	16 (15.4%)	11 (20.0%)
Heart failure	Yes	8 (7.7%)	2 (3.6%)
Cerebrovascular disease	Yes	3 (2.9%)	1 (1.8%)
Hypothyroidism	Yes	15 (14.4%)	9 (16.4%)
Glaucoma	Yes	1 (1.0%)	3 (5.5%)
Takes ipratropium	Yes	1 (1.0%)	1 (1.8%)
Takes beta-blocker	Yes	38 (36.5%)	23 (41.8%)
Takes ARB	Yes	43 (41.3%)	20 (36.4%)
Takes ACE	Yes	15 (14.4%)	5 (9.1%)
Takes CCB	Yes	15 (14.4%)	5 (9.1%)
Takes antidepressants	Yes	8 (7.7%)	2 (3.6%)
Takes amiodarone	Yes	1 (1.0%)	1 (1.8%)
Dexmedetomidine induction	Yes	10 (9.6%)	7 (12.7%)
Sevoflurane maintenance	Yes	30 (28.8%)	21 (38.2%)
Desflurane maintenance	Yes	26 (25.0%)	11 (20.0%)
Propofol maintenance	Yes	77 (74.0%)	37 (67.3%)
Remifentanil maintenance	Yes	90 (86.5%)	45 (81.8%)
Outcome: Intraoperative bradycardia	Yes	21 (20.2%)	20 (36.4%)

There was a roughly equal number of males and females, while the overwhelming majority of patients had an ASA-PS score of 3. Roughly two-thirds (65.4%) of the patients underwent unilateral MRND. Approximately one quarter (25.8%) of all patients in our sample developed bradycardia intraoperatively. No patients developed asystole or cardiac arrest.

Table 2 follows with descriptive statistics for the quantitative exposure variables. No patient was initially bradycardic (PR < 50). Most data were not normally distributed; however, this did not have any bearing on the logistic regression models discussed below.

**Table 2:** Summary measures for all quantitative data in patients undergoing unilateral or bilateral modified radical neck dissection (MRND). For normally distributed data, we report mean (SD). For non-normally distributed data, we report median (IQR (min–max)).

Variable	Unilateral MRND, n = 104	Test for normality	Bilateral MRND, n = 55	Test for normality
Age (y)	70 (62–76 [37–88])	<0.001	73 (63–77 [35–86])	0.003
BMI (kg/m^2^)	27 (25–28 [18–42])	<0.001	27 (25–29 [20–42])	0.002
Initial SBP (mmHg)	138 (125–151 [107–180])	0.012	138 (21.8)	0.268
Initial DBP (mmHg)	78 (12)	0.07	79 (11)	0.051
Initial PR (bpm)	74 (69–78 [51–105])	<0.001	76 (11)	0.087
Total fentanyl administered (mg)	0.65 (0.50–1.0 [0.20–2.0])	<0.001	1.0 (0.50–1.4 [0.25–2.0])	<0.001
Surgical time (min)	240 (186–300 [60–670])	<0.001	405 (300–640 [180–730])	0.001
Anaesthetic time (min)	280 (240–345 [90–720])	<0.001	465 (338–710 [150–780])	0.003

Simple univariate models

We then developed simple univariate models as described in the methods section. Table 3 shows measures of association for the relationship between categorical variables and the outcome, but only in those cases with a p-value below 0.2.

**Table 3:** Simple univariate anlayses for the association between exposure variables and intraoperative bradycardia. ref.: reference group.

Exposure variable	Proportion with bradycardia (%)	Odds ratio (95% C.I.)	P-value	Relative risk
MRND laterality				
Unilateral	20.2	0.443 (0.214, 0.918)	0.027	0.555 (0.331, 0.932)
Bilateral	36.4	ref.		ref.
ASA-PS		N/A	<0.001	N/A
2	0.0			
3	22.3			
4	66.7			
Hypertension				
Yes	29.4	1.74 (0.796, 3.82)	0.162	1.52 (0.828, 2.81)
No	19.3	ref.		ref.
Atrial fibrillation				
Yes	66.7	9.48 (3.79, 23.7)	<0.001	3.83 (2.42, 6.04)
No	17.4	ref.		ref.
Takes beta-blocker				
Yes	36.1	2.35 (1.14, 4.84)	0.019	1.86 (1.10, 3.14)
No	19.4	ref.		ref.
Dexmedetomidine induction				
Yes	52.9	3.87 (1.38, 10.8)	0.007	2.35 (1.37, 4.04)
No	22.5	ref.		ref.

Among the quantitative exposure variables, only the total fentanyl dose was found to be significantly associated with intraoperative bradycardia: the OR for each additional mg of fentanyl was 2.91 (95% C.I.: 1.35-6.20) with a p-value of 0.006.

Multiple univariate models

Using multiple logistic regression, we constructed a model with all the variables in Table 3 as well as the total fentanyl dose. Variables with a non-statistically significant association were removed in a stepwise fashion, starting with the largest p-value. Only the main exposure variable (MRND laterality) was retained regardless of the significance of its association with the outcome. The resulting model can be seen in Table 4.

**Table 4:** Multiple univariate analysis for the association between exposure variables and intraoperative bradycardia. ref.: reference group; N/C: non-calculable.

Outcome	Intraoperative bradycardia		
Exposure variable	Adjusted odds ratio (aOR)	95% C.I.	P-value
MRND laterality			
Unilateral	0.461	0.192-1.11	0.082
Bilateral	ref.		
ASA-PS			
2	N/C	N/C	0.999
3	0.125	0.0340-0.457	0.002*
4	ref.		
Atrial fibrillation			
Yes	11.4	4.10-31.8	<0.001*
No	ref.		
Dexmedetomidine induction			
Yes	4.57	1.34-15.6	0.015*
No	ref.		
Intercept value: 1.73			0.403

## Discussion

Given the lack of clinical literature on bradycardia in neck dissection, this study represents a unique addition to the body of knowledge regarding this topic. The factors identified as having a statistically significant correlation with the development of intraoperative bradycardia in neck dissection were an ASA-PS classification score of 4, the presence of AF, and the administration of dexmedetomidine for induction. We proceed to discuss these one by one.

There was a very significant increase in the adjusted odds of bradycardia for ASA-PS 4 compared to ASA-PS 3. In conjunction with the results of the simple univariate analysis, it seems that, in terms of RR, a change from ASA-PS 3 to ASA-PS 4 is associated with roughly triple the risk. Unfortunately, due to the very small number of patients with ASA-PS 2, it was not possible to calculate the adjusted OR (aOR) in relation to this group. We suspect that this would either have similar adjusted odds to ASA-PS 3 or even more reduced adjusted odds. Besides, ASA-PS classification is and independent predictor of intraoperative complications [8].

The strongest association, as well as the most statistically significant one, was the presence of AF. In the simple univariate analysis, it was associated with roughly four times the risk of intraoperative bradycardia compared to patients without AF. Given that the aOR is even larger than the unadjusted one, it is safe to say that the adjusted RR is similar to the unadjusted one. Patients with AF are on antiarrhythmic drugs which may precipitate bradycardia during opiod [9] or propofol [10] administration. Overall, a recent study [11] suggests that pre-existing AF is associated with adverse postoperative outcomes after non-cardiac surgery.

The administration of dexmedetomidine was found to have a fairly strong association, since it more than doubled the risk in the simple univariate analysis, while the strength of the association is maintained in the multiple univariate models. Dexmedetomidine was administered intravenously only to patients undergoing awake fibreoptic intubation [12]. The administration was ceased after successful intubation. In these patients, bradycardia was not recorded during the administration of dexmedetomidine, but well after dexmedetomidine had been discontinued and surgical manipulations had begun. Nonetheless, bradycardia occurrence may be related to dexmedetomidine administration, on the grounds of its pharmacokinetics, a phenomenon that warrants further investigation [13].

MRND laterality was found to be statistically significant in the simple analysis but not in the adjusted model. It was nevertheless very close to statistical significance throughout the variable elimination process; therefore, we suspect that the power of the study may have been insufficient to identify a significant correlation. Whether this is indeed a false negative could be determined in the future by a study with a bigger sample size. Up to our knowledge, there are no reports relating neck dissection laterality with the occurrence of bradycardia during neck dissection surgeries.

Taking a regular beta-blocker was another exposure variable with a statistically significant effect in the simple model which did not remain significant in the adjusted model. However, unlike MRND laterality, we suspect that the reason for this is biological rather than statistical: the correlation of beta-blockers with intraoperative bradycardia was confounded by the fact that several patients who take beta-blockers (about one third in this cohort) do so because of AF - a factor with a significant effect on intraoperative bradycardia, as discussed above. Overall, elder patients (>65 years) on beta-blockers undergoing major surgery are likely to present intraoperative bradycardia [14].

With regard to local lidocaine in the carotid bulb, which was not performed in any of the operations that we observed. Infiltration of the carotid bulb is not a universally accepted technique [15].

Among the limitations of this study, we have identified the following: we did not take into account more specific aspects of the surgical procedure, such as the effect of left vs. right unilateral MRND and the extent of lymphatic tissue clearance (which and how many levels). More importantly, though, we did not take into account whether bradycardia was temporally associated with manipulation of the carotid bulb by the surgeon or whether a cessation of the surgical stimulus had any effect on the heart rate.

## Conclusions

MRND laterality was close to statistical significance. Bigger sample sizes may provide more definitive information, since the effect of MRND laterality on intraoperative bradycardia remains unclear. Our findings can inform clinical practice so that clinicians know when to expect bradycardia and are better prepared to manage it.
